# Research on Bending Performance of Three-Dimensional Deep Angle Interlock Kevlar/EP Armor Material

**DOI:** 10.3390/ma15155321

**Published:** 2022-08-02

**Authors:** Jianhua Zheng, Lin Zhong, Hongxia Chen, Xiaomei Huang, Haijian Cao

**Affiliations:** 1 School of Textile and Clothing, Nantong University, Nantong 226007, China; 1515032079@ntu.edu.cn (J.Z.); 18136523883@163.com (L.Z.); h.xmei@ntu.edu.cn (X.H.); 2 School of Textile Science and Engineering, Jiangnan University, Wuxi 214126, China

**Keywords:** composite armor material, laying method, resin content, weft density, number of stacking layers

## Abstract

Three-dimensional (3D) woven composites have attracted much attention in the lightweight research of protective armor due to their high specific strength and good impact resistance. However, there are still many gaps in terms of the performance and influencing factors of three-dimensional deep-angle-interlock (3DDAI) Kevlar/EP armor materials. Therefore, in order to prepare 3DDAI Kevlar/EP armor materials with excellent ballistic resistance and mechanical properties, this paper studies the bending performance of 3DDAI Kevlar/EP armor materials and the influence of the number of stacking layers, resin content, laying method, and weft density. Finally, we compare it with the traditional two-dimensional (2D) plain laminated Kevlar/EP armor material. The results showed that when the 3DDAI Kevlar/EP armor material was subjected to bending load, the upper and bottom layers of the material had a great influence on the initial stiffness and fracture strength of the material, respectively; when the material’s warp and weft density are quite different, the utilization rate of the yarn and the strength of the material are negatively affected; the fracture energy of the 3DDAI Kevlar/EP armor material prepared by the orthogonal laying method was about 20% higher than that of the 3DDAI Kevlar/EP armor material with the unidirectional layering method; and the bending performance of the 3DDAI Kevlar/EP armor material in the weft direction was better than that of the 2D plain laminated Kevlar/EP armor material, with the 3DDAI Kevlar/EP armor material having better delamination resistance. The research results will lay the foundation for structural optimization and engineering applications of such materials.

## 1. Introduction

Fiber-reinforced composite materials are widely used in aerospace, transportation, military protection, and other fields due to their low density and high strength [1,2,3]. In the field of protection, fiber-reinforced composite materials can be directly prepared into armor for protection and can also be used as a back plate to form composite armor with a ceramic/metal panel [4,5].

Three-dimensional (3D) woven composite materials outperform the traditional two-dimensional (2D) fabrics laminated to prepare laminate armor [5], in terms of impact resistance and interlayer shear resistance [6,7,8,9]. Due to the designability and structural complexity of 3D woven composites, previous studies have mainly focused on fabric structure, hybrid effects, and failure mechanisms. Warren et al. [10] combined a digital image method to study the tensile, compression, and in-plane shear performance of 3D orthogonal, shallow bend-interlock composite materials and compared them with 2D woven composites. Dai et al. [11] studied the tensile, compression, and bending properties of four 3D orthogonal and two 3D deep-angle-interlock composite materials, and found that the initial damage positions of the six composite materials with different structures were all at the interweaving point of the resin-rich zone. Zhang et al. [12] used immersion-focused ultrasound imaging and micro-computed tomography technology to study the bending properties and damage mechanism of 3D shallow bend-interlock carbon/epoxy composites along different angles (0°, 30°, 45°, 90°). The study found that the material has higher flexural strength, initial modulus, and lower bending deformation along the axis (0°, 90°) than the off-axis (30°, 45°). The on-axis sample exhibits quasi-brittleness, and the off-axis style exhibits better elongation properties and effectively reduces cracks and delamination. Fan et al. [13] prepared 3D orthogonal C-G/epoxy hybrid composites, with glass fibers in the upper and bottom layers and carbon fibers in the middle layer, and Z-binder yarns to study their bending properties and bending fatigue properties and compared them with the 3D orthogonal carbon fiber/epoxy composites and the laminated C-G/epoxy hybrid composites. The study found that the strength and modulus of the 3D C-G hybrid composites were higher than that of the laminated C-G hybrid composites, and the modulus of the 3D C-G hybrid composites was lower than that of the 3D carbon fiber composites, but the failure strain and strength were higher than those of the 3D carbon fiber composites. The study also found that the fracture origin of the 3D C-G hybrid composites and 3D carbon fiber composites in static bending was from the kinking failure and shearing failure of the top layer. The compression damage of warp and Z-binder yarns was the main failure mode of fatigue testing. Guo et al. [14] studied the effect of weaving parameters on the mechanical properties of 3D multi-angle interlocking woven composite materials and found that weaving parameters have a greater influence on the failure morphology of the material. Zheng et al. [4] established a mesoscopic model of 3D shallow bend-interlock composite materials, based on numerical simulations, to study the damage mechanism of the material and conducted tensile experiments on the material to verify the accuracy of the simulation. For 3D angle-interlock structural materials, the current research on bulletproof ability mainly focuses on the fabric level, as illustrated in the following studies. Yang et al. [15] conducted a finite element simulation of the ballistic performance of 3D angle-interlock fabrics and 2D plain laminated fabrics, and found that the 3D angle-interlock fabric ratio capacity absorbs higher than the 2D plain laminated fabric; they also found that 3D fabrics have better surface diffusion capacity. Wei et al. [16] conducted ballistic impact experiments on 3D deep-angle-interlock fabrics, which revealed the impact damage evolution, energy absorption mechanism, and stress wave distribution of 3D deep-angle-interlock fabrics.

Due to the limitations of weaving technology, the thickness of a single 3D fabric is often limited to 1–6 mm [17,18,19], and the preparation of composite materials is still unable to reach a high level of ballistic protection. Min et al. [20] analyzed the ballistic performance of 3D deep-angle-interlock stacking layer armored materials of different structures from the aspects of damage depth, scale-out, damage volume, etc., and found that the bullet-proof effect of the five-layer deep-angle-interlock structure is better than that of the four-layer deep-angle-interlock structure. However, the effect on the mechanical and ballistic performance of the 3D deep-angle-interlock armored materials has not been sufficiently studied, such as the series of fabric structure parameters, fabric stacking direction, and resin content.

Therefore, to prepare three-dimensional deep-angle-interlock (3DDAI) Kevlar/EP armor materials with excellent mechanical and ballistic properties, in this paper, 3DDAI Kevlar fabric was used as the reinforcement and epoxy resin was used as the matrix to study the influence of the bending properties. These include the number of stacking layers, resin content, fabric weft density, and laying method. The work presented in this study lays a foundation for subsequent research on the ballistic resistance of 3DDAI Kevlar/EP armor materials.

## 2. Experiment

### 2.1. Materials and Equipment

**Figure 1:**
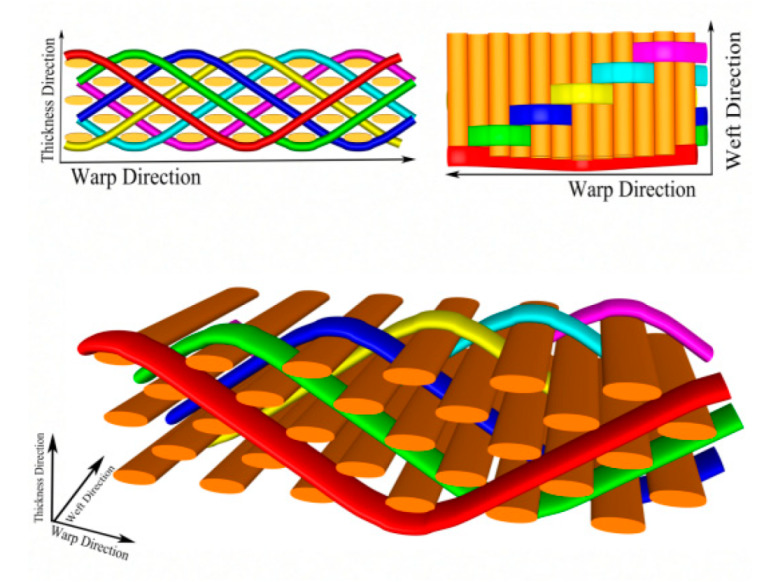
Schematic diagram of 3DDAI fabric.

The 3D deep-angle-interlock Kevlar fabrics (three different weft densities, as shown in Table 1), were self-made, as shown in Figure 1; Kevlar plain fabrics were supplied by Yantai Taihe New Material Co., Ltd. (Yantai, China); Epoxy resin E-51 was supplied by Nantong Xingchen Synthetic Material Co., Ltd. (Nantong, China); Polyetheramine D230 curing agent was supplied by Changzhou Runxiang Chemical Co., Ltd. (Changzhou, China); 101A-4S electric heating blast-drying oven was supplied by Nanjing Wohuan Technology Industrial Co., Ltd. (Nanjing, China); WG-1200 multifunctional ceramic tile cutting machine was supplied by Sichuan Wanguang Machinery Equipment Co., Ltd. (Guanghan, China); Instron 5969H universal material testing machine was supplied by Instron Testing Equipment Trading Co., Ltd. (Shanghai, China); LEICASAPO stereo microscope was supplied by Leica Microsystems Trading Co., Ltd. (Shanghai, China).

### 2.2. Sample Preparation

We utilized the 300 mm × 300 mm 3D deep-angle-interlock Kevlar fabric as the reinforcement. Epoxy resin E-51 and curing agent polyetheramine D230 were mixed uniformly in the ratio of 4:1 as the matrix, and the vacuum-assisted molding process was used to compound. The material was cut by the WG-1200 multifunctional ceramic tile cutting machine, according to the experimental requirements, and the parameters of the 3DDAI Kevlar/EP armor material sample are listed in Table 2.

The resin content/fiber volume fraction has an important influence on the mechanical properties of the material [21,22,23], so it is necessary to control the resin content during the material preparation process. Among them, the calculation formula for resin content and fiber volume fraction is as follows:

(1) $$M_{m}=\frac{1-m_{f}}{m_{c}}$$

(2) $$V_{f}=\frac{m_{f}/\rho_{f}}{V_{c}}$$

where *M_m_* is the resin content. *m_f_* is the fiber quality. *m_c_* is the composite material quality. *V_f_* is the fiber volume fraction. *V_c_* is the volume of composite *ρ_f_* is the density of fiber.

### 2.3. Bending Test

The bending performance of the 3DDAI Kevlar/EP armor material was tested on the Instron 5969 H universal material testing machine according to GB/T1449-2005 [24]. The ratio of the bending span to the thickness of the pattern was 16:1. The bending test speed was 2 mm/min. The preload was set to 3–8 N, and the load continues until the pattern fails. In order to ensure the validity of the data, each data-point was tested at least five times, and the average value was taken.

The bending stress was calculated using Equation (3); the bending strain was calculated using Equation (4); and the bending modulus was calculated using Equation (5).

(3) $$\sigma =3P\cdot l/2b\cdot h^{2}$$

(4) $$\varepsilon =6S\cdot h/l^{2}$$

(5) $$E=\frac{l^{3}\cdot \Delta P}{4\cdot b\cdot h^{3}\cdot \Delta S}$$

where *σ* is the bending stress. *P* is the bending load. *l* is the bending span. *b* is the sample width. *h* is the sample thickness. *ε* is the bending strain. *S* is the bending deflection. Δ*P* and Δ*S* are the load increments of the initial line segment and the displacement increment of the middle point of the span, respectively.

## 3. Results and Discussions

### 3.1. Effect of Stacking Layers of 3DDAI Kevlar Fabrics on Bending Properties

The 3DDAI fabric with a weft density of 50 picks/cm was used as the reinforcement, and armor materials (resin content 44% ± 1%) of different fabric pieces were prepared through the superimposition of the unidirectional laying method, including 3DDAI Kevlar/EP armor materials made of single, two, three, and four fabric laminates. The warp and weft directions of the material were tested for bending performance, respectively.

#### 3.1.1. Bending Stress-Strain Curves

**Figure 2:**
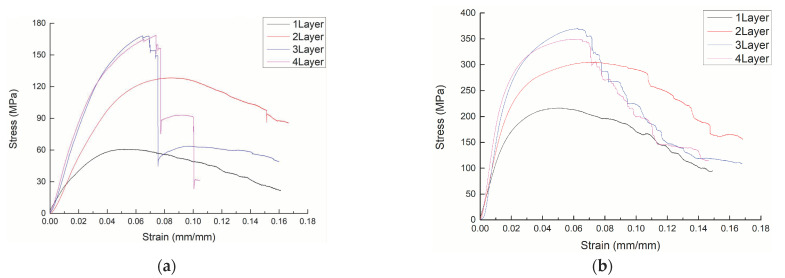
The bending stress-strain curve of 3DDAI Kevlar/EP armor materials with different stacking layers. (**a**) The warp direction. (**b**) The weft direction.

The bending stress-strain curves of 3DDAI Kevlar/EP armor materials with different fabric layers are shown in Figure 2.

**Figure 3:**
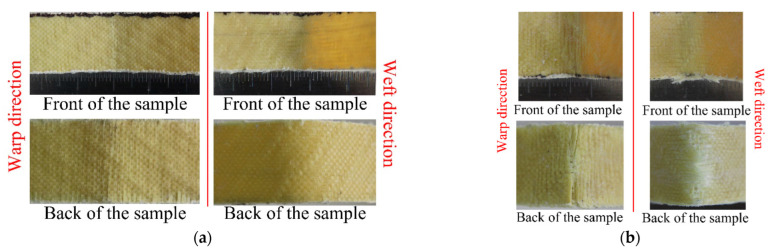
Bending failure morphologies of 3DDAI of Kevlar/EP armor materials with 1 to 2 layers. (**a**) 1 layer. (**b**) 2 layer.

It can be seen from Figure 2 that 3DDAI Kevlar/EP armor materials with different stacking layers have different bending stress-strain responses. Among them, the curve characteristics of 1 to 2 layers are similar. The stress first increases linearly with the strain, then increases nonlinearly, and then decreases slowly after reaching the maximum value. This is because Kevlar fibers have good toughness, and the material failure is mainly due to the decrease in stiffness caused by buckling deformation. The damage morphology of 1–2 layers of 3DDAI Kevlar/EP armor materials is shown in Figure 3. When there was only one layer of fabric, the yarn did not break; when there were two layers of fabric, the local yarn broke. The 3 to 4 layer bending curve characteristics are similar and are described as follows: in the initial stage, the stress increases linearly with the strain; subsequently, the stress increases nonlinearly with the strain; and then, the stress reaches its maximum value, and the material fails. Finally, as the strain increases further, the stress drops sharply.

**Figure 4:**
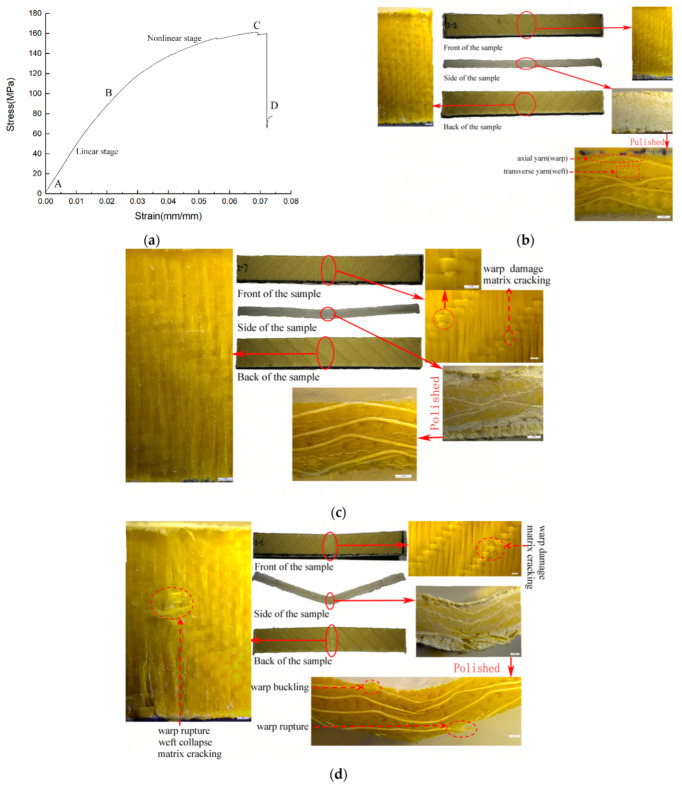
The bending response of 3DDAI Kevlar/EP armor material when loaded along the warp direction. (**a**) The bending stress-strain curve. (**b**) The topography of the material at point B. (**c**) The topography of the material at point C. (**d**) The topography of the material at point D.

**Figure 5:**
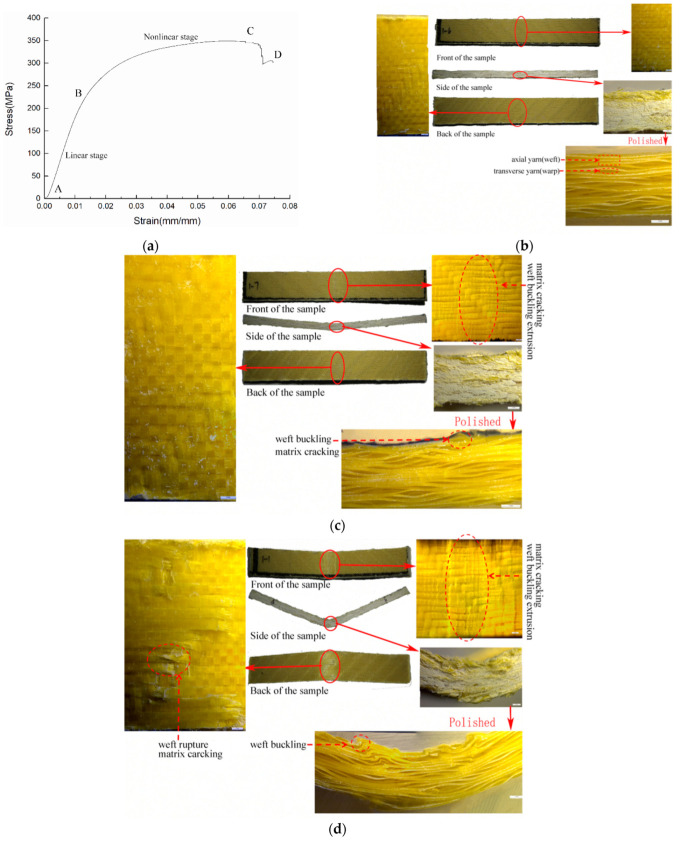
The bending response of 3DDAI Kevlar/EP armor material when loaded along the weft direction. (**a**) The bending stress-strain curve. (**b**) The topography of the material at point B. (**c**) The topography of the material at point C. (**d**) The topography of the material at point D.

In order to further study the bending characteristics of multilayer 3DDAI armor materials, taking 4 layers of 3DDI armor materials as an example, the damage morphology was analyzed by a LEICASAPO stereo microscope. Among them, the slope of the curve was calculated with a strain of 0.002 mm/mm as the limit, and the test was stopped at the point of sudden change of the slope to get the failure morphology of the material at different stages, which is shown in Figure 4 and Figure 5. In these pictures, we noticed that the B and C points when the material was loaded in the weft direction come earlier than when the material was loaded in the warp direction. This is because the buckled warp yarn in the material has a higher failure strain than the straightened weft yarn. Among them, there was no obvious damage to the material in the linear growth phase (AB section, A is the curve’s starting point). In the non-linear growth stage (the BC section), the axial yarns in the upper layer of the material accumulate damage, and the matrix is broken. When loaded in the warp direction, it shows warp yarn damage and matrix fragmentation around the interweaving point on the upper surface of the material, as shown in Figure 4c. When loaded in the weft direction, it shows buckling and extrusion of the weft yarn in the upper layer of the material and local cracking of the matrix, as shown in Figure 5c. The ultimate failure of the material (point D) was caused by the fracture of the axial yarns in the bottom layer of the material. When loaded in the warp direction, it appears that the underside of the material has been broken, and the weft yarns have collapsed. The bottom warp yarns located directly under the upper indenter broke first and then spread in the thickness direction, showing longitudinal cracks as shown in Figure 4d. When loaded in the weft direction, it appears that the material’s bottom layer matrix was broken. The bottom weft yarn, located directly under the upper indenter, breaks first and then expands in the plane, resulting in a diagonal breaking path of the weft yarn along the weaving point area, as shown in Figure 5d.

#### 3.1.2. Bending Properties

**Figure 6:**
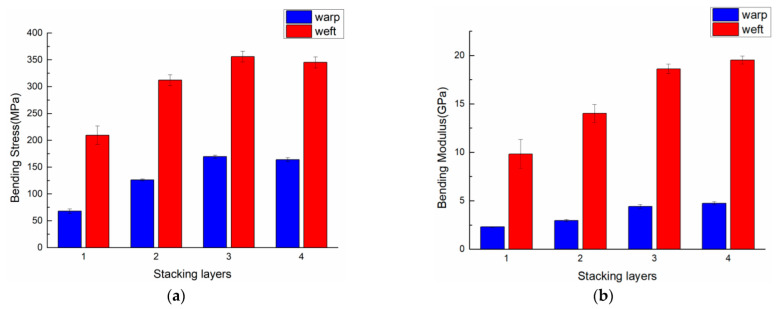
Comparison of the bending properties of materials with different stacking layers. (**a**) Bending strength. (**b**) Bending stiffness.

The bending properties of the 3DDAI Kevlar/EP armor materials with different superimposed layers are listed in Table 3. In order to more intuitively evaluate the influence of different stacking layers on the bending properties of materials, the bending performance comparison of different stacking layers of materials is shown in Figure 6.

It can be seen from Table 3 that: (1) The bending performance in the weft direction was significantly better than that in the warp direction. (2) When the number of stacked layers reaches 3 layers, the bending strength was the largest (169.4 MPa along the warp direction and 356 MPa along the weft direction). (3) When reaching 4 layers, the bending modulus was the largest (in the warp direction: 5.75 GPa, along the weft direction: 19.52 GPa).

It can be seen from Figure 6 that the bending strength of the 3DDAI Kevlar/EP armor material first increases with the number of fabric stacking layers, reaches a peak when the number of stacking layers reaches three, and then decreases. According to the bending strength formula, the bending strength of the material increases as a logarithmic function with an increase in thickness. However, in George J. Dvorak’s study [25], it was found that with the increase in the thickness of the laminate layer, the strength of the matrix in the material decreased. Therefore, when the number of 3DDAI Kevlar/EP armored material overlays increases to a certain extent, the increase in bending strength is weaker than the decrease in matrix strength, resulting in a decrease in the overall strength of the material. The increase in the number of stacking layers in the 3DDAI Kevlar/EP armor material and the increase in the total amount of fibers involved in resisting deformation shows that the bending modulus of the material increases. However, the increase in bending module decreases with the increase in the number of stacking layers due to the material’s resistance to deformation by synergy between different layers, rather than the participation of all areas of the material itself in resisting deformation.

### 3.2. Effect of Epoxy Resin Content on Bending Properties

The 3DDAI fabric with a weft density of 50 picks/cm was the reinforcement, and the armor material, with different contents of epoxy resin, was prepared by the symmetrical laying method, including resin contents of 34.27%, 36.75%, 40.39%, 44.61%, and 48.59% of the 3DDAI Kevlar/EP armor materials were tested in the warp and weft directions, respectively.

#### 3.2.1. Bending Stress-Strain Curves

**Figure 7:**
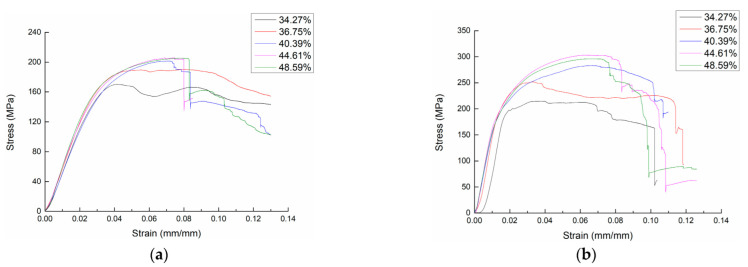
The bending stress-strain curve of 3DDAI Kevlar/EP armor materials with different contents of epoxy resin. (**a**) The warp direction. (**b**) The weft direction.

Figure 7 shows the bending stress-strain curves of 3DDAI Kevlar/EP armor materials with different resin contents. In the figure, we found that the stress-strain curve of the material was more unstable when the resin content was 34.27% (fiber volume content of 54.92%) and 36.75% (52.71%), which may be because the fabric structure of 3DDAI was relatively loose and low resin content will result in more voids inside the material and thus an unstable response of the material during the bending process. When the resin content was 40.39% (48.94%), 44.61% (46.12%), and 48.59% (43.38%), the stress-strain curve of the material was relatively stable, indicating that the resin content in this range can prepare 3DDAI Kevlar/EP armor material with stable performance.

#### 3.2.2. Bending Properties

**Figure 8:**
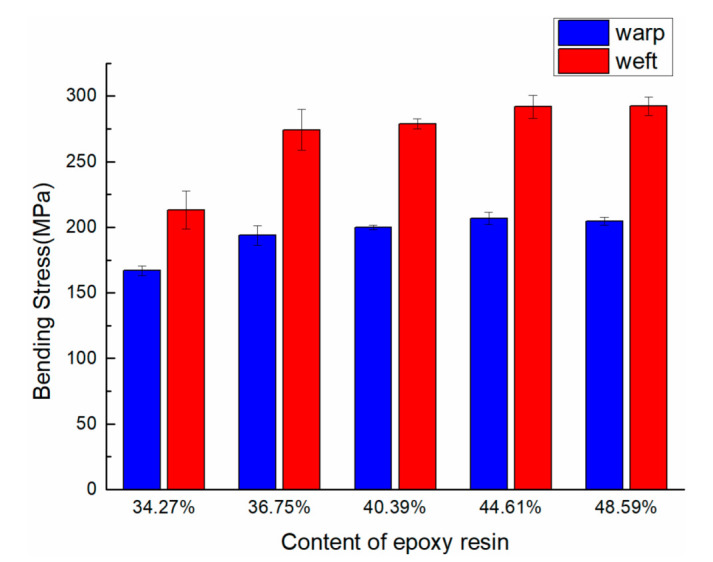
Comparison of the bending strength of materials with different contents of epoxy resin.

The bending properties of 3DDAI Kevlar/EP armor materials with different resin content are listed in Table 4. In order to evaluate the influence of different resin contents on the bending properties of the material, the bending properties comparison of the material with different resin contents is shown in Figure 8.

As can be seen from Table 4, the resin content of the 3DDAI Kevlar/EP armor material’s bending strength was the highest at 44.61% (along the warp direction: 206.8 MPa, along the weft direction: 307 MPa), and the bending strength was the lowest when the resin content was 34.27% (along the warp direction: 167 MPa, along the weft direction: 216.4 MPa).

As can be seen from Figure 8, the bending strength of the material increases with the increase of the resin content when the resin content is increased from 34.27% to 44.61%. When the resin content of the material is 44.61% to 48.59%, the bending strength decreases with the increase of the epoxy resin content, but the degree of the decrease is not very obvious. This is because if the resin content is too low, the matrix will be unable to play a useful role in load transmission and reducing the synergy between fibers and the matrix will result in the lower material’s strength. However, if the resin content is excessively high, the material strength will decrease too, due to the relative reduction of fiber as the carrier main body [26,27]. Therefore, in the armor material preparation process, it could improve the utilization rate of the material by controlling the appropriate resin content, and the optimal resin content of the 3DDAI Kevlar/EP armor material should be controlled within the range of 40% to 49%.

### 3.3. The Effect of Laying Method on Bending Properties

In the literature on woven and woven-composite ballistic materials, it was found that the ballistic performance of quasi-isotropic materials in the macroscopic plane was better than that of anisotropic materials in the macroscopic plane [28,29,30]. Therefore, the following 3DDAI Kevlar/EP armor materials with different laying methods were designed as far as possible to be macro-quasi-isotropic except for the unidirectional laying materials.

**Figure 9:**
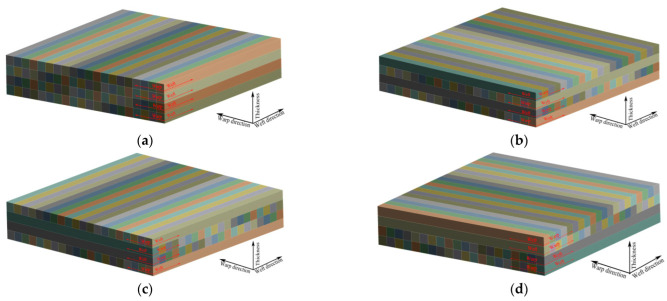
Schematic diagram of different laying methods. (**a**) Unidirectional laying method. (**b**) Orthogonal laying method. (**c**) Symmetrical laying method. (**d**) 2/2 laying method.

The 3DDAI fabric, with a weft density of 50 picks/cm, was used as the reinforcement and was combined with the resin system to prepare armor materials with different laying methods. 3DDAI Kevlar/EP armor materials (resin content 44% ± 1%) was prepared using the unidirectional laying method, the orthogonal laying method, the symmetrical laying method, and the 2/2 laying method, and bending performance tests were conducted along the warp and weft directions, respectively. Figure 9 is a schematic diagram of different laying methods.

#### 3.3.1. Bending Stress-Strain Curves

**Figure 10:**
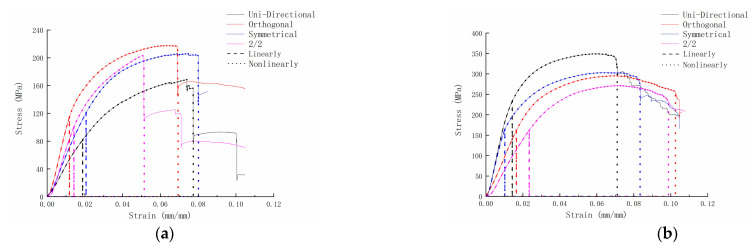
The bending stress-strain curve of 3DDAI Kevlar/EP armor materials with different laying methods. (**a**) The warp direction. (**b**) The weft direction.

Figure 10 is the bending stress-strain curve of the 3DDAI Kevlar/EP armor materials with different laying methods. From the figure, we can see that the materials with different laying methods have similar bending characteristics. However, the response of each stage was different, so the fracture energy of the material obtained by calculating the curve area of each stage is listed in Table 5.

From Table 5, we can observe that: (1) The nonlinear phase of the material lasts longer and can absorb more energy. (2) In general, the order of fracture energy was orthogonal laying method > symmetric laying method > unidirectional laying method > 2/2 laying method. Furthermore, the fracture energy of the material of the orthogonal layer was about 20% higher than that of the material of the unidirectional layer. (3) The fracture energy of the material whose bottom layer was in the weft direction (90°) was significantly higher than that of the material whose bottom layer was in the warp direction (0°). (4) The material whose upper layer was in the warp direction shows higher ultimate strain at each stage than the material whose upper layer was in the weft direction.

#### 3.3.2. Bending Properties

The bending properties of the 3DDAI Kevlar/EP armor materials with different laying methods are listed in Table 6. It can be seen from the table that: (1) In the unidirectional and symmetrical laying methods, the bending strength and bending modulus of the armor material along the weft direction were far greater than the bending strength and bending modulus of the material along the warp direction. (2) The bending strength of the armor material along the weft direction was greater than the bending strength of the material along the warp direction in the orthogonal and 2/2 laying methods, and the bending modulus of the material in the warp direction was slightly greater than the bending modulus of the material in the weft direction. (3) The bending properties of the 3DDAI Kevlar/EP armor materials with different laying methods were different, and the bending performance of the materials along the weft direction was in the order of unidirectional laying method > symmetrical laying method > orthogonal laying method > 2/2 laying method; the bending performance of the materials along the warp direction was in the order of orthogonal laying method > 2/2 laying method > symmetrical laying method > unidirectional laying method. (4) When the 3DDAI Kevlar/EP armor material was subjected to bending load, the upper and bottom layers of the material were the main bodies that bore the load, and the stiffness contribution of the upper layer was greater than the bottom layer; however, the strength contribution of the bottom layer was greater than the upper layer.

**Figure 11:**
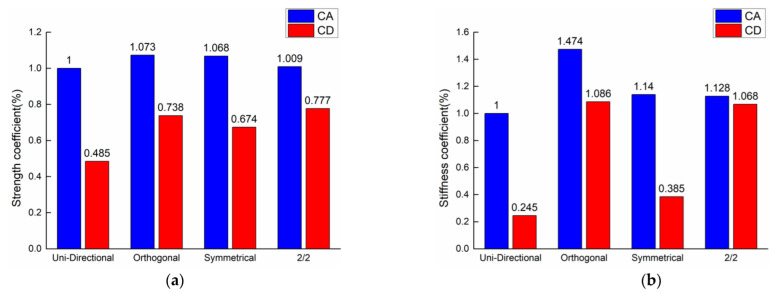
Comparison of the bending performance of materials with different laying method. (**a**) Strength coefficient. (**b**) Stiffness coefficient.

In order to make the material appear quasi-isotropic in the macroscopic plane, the mechanical properties of the material in different directions are improved by changing the laying method. However, as was described above, there were still anisotropic features in the material when bearing the bending load after changing the method of laying. Therefore, we used Equation (6), the Coefficient of Ascension (CA), to express the comprehensive bending performance of the material relative to the unidirectional laying material, and the difference between the bending properties of the material in different directions was expressed by Equation (7), the Coefficient of Difference (CD; the closer to 1, the smaller the difference). A comparison of the bending coefficients of the 3DDAI Kevlar and EP armor materials in different laying methods was made, as shown in Figure 11.

(6) $$Q_{CA}=(Q_{1}/Q_{warp}+Q_{2}/Q_{weft})\cdot 1/2$$

(7) $$Q_{CD}=Q_{1}/Q_{2}$$

where *Q_CA_* is the Coefficient of ascension, *Q_CD_* is the coefficient of difference, *Q*_1_ is the bending performance of the material along the warp direction, *Q*_2_ is the bending performance of the material along the weft direction, *Q_warp_* is the bending performance of the unidirectional laying material along the warp direction, *Q_weft_* is the bending performance of the unidirectional laying material along the weft direction.

As shown in Figure 11, after changing the laying method, the comprehensive bending performance of the 3DDAI Kevlar/EP armor material was improved. Among these changes, the comprehensive bending performance improvement of the orthogonal laying material was the highest. The difference in bending performance between 2/2 laying materials in different directions was the smallest, and the difference in bending performance of orthogonal laying materials was also very small. In summary, the preparation of 3DDAI Kevlar/EP armor material by the orthogonal laying method is beneficial to maximize the potential to improve the comprehensive bending performance of the material, while reducing the difference in bending performance between different directions of the material.

### 3.4. The Effect of Fabric Weft Density on Bending Properties

As noted above, the preparation of 3DDAI Kevlar/EP armor materials in an orthogonal laying method was conducive to maximizing the potential to improve the comprehensive bending performance of the material, while reducing the difference in bending performance between different directions of the material. Therefore, armor materials were mainly prepared by the orthogonal laying method to study the effect of different fabric weft densities on the bending properties of the materials.

**Figure 12:**
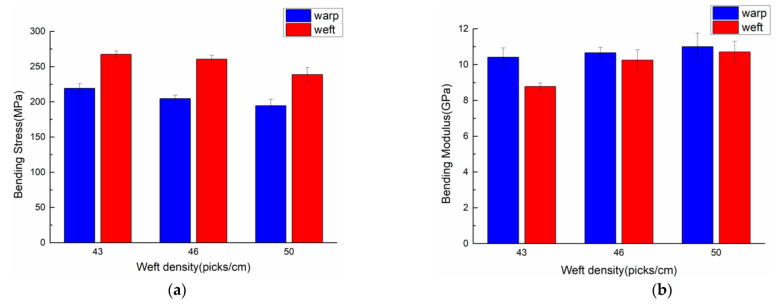
Comparison of the bending properties of materials with different weft densities. (**a**) Bending strength. (**b**) Bending stiffness.

The 3DDAI fabrics with weft densities of 43, 46, and 50 picks/cm were used as reinforcements to prepare armor materials (resin content 46% ± 1%), and bending performance tests were carried out along the material’s warp and weft direction. The bending properties of the 3DDAI Kevlar/EP armor materials with different weft densities are listed in Table 7. In order to more intuitively evaluate the effect of different fabric weft densities on the bending performance of the material, the comparison of the bending performances of different fabric weft densities is shown in Figure 12.

As can be seen from Table 7, the 3DDAI Kevlar/EP armor material has the highest bending strength at 43 picks/cm of weft density (along warp direction: 220.8 MPa, along weft direction: 267.6 MPa), and the lowest bending modulus (along warp direction: 10.42 GPa, along weft direction: 8.81 GPa); when the weft density of the fabric was 50 picks/cm, the bending strength was the lowest (along the warp direction: 194.6 MPa, along the weft direction: 238.7 MPa), and the bending modulus was the highest (along the warp direction: 11 GPa, along the weft direction: 10.71 GPa).

As can be seen from Figure 12, when the weft density of the fabric increases from 43 picks/cm to 50 picks/cm, the bending strength of the material decreases with the increase of the fabric weft density. The internal voids of the fabric are reduced when the fabric weft density is increased, and the reason why the material can macroscopically ensure the same resin content is that more resin accumulates on the surface of the material to form a “resin-rich zone,” which means the material with the higher fabric weft density actually has less resin content in the interior than the material with the lower fabric weft density. Firstly, this is because the increase in the weft density causes the “lean resin zone” in the material to be more prone to cracks. Secondly, due to the increase in the weft density, the degree of squeezing inside the yarn increases and the internal stress increases, resulting in a decrease in the overall bending strength of the material; these results were also found in the study of HA Aisyah [31]. The bending modulus of the material increased as the fabric weft density increased from 43 picks/cm to 50 picks/cm, and the increase in the bending modulus of the material along the weft direction is greater than that of the material along the warp direction. This is owing to the fact that when the material is subjected to bending load, the upper layer bears the axial compressive loading while the bottom layer bears the axial tensile loading [32]. With an increase in fabric weft density, the axial fibers along the weft direction increase and the transverse fibers along the warp direction rise. On the other hand, the axial mechanical properties of the fibers far outperform the transverse mechanical properties [33]. For the reasons stated above, the increase in the bending modulus of the material along the weft direction is greater than the rise in the bending modulus of the material along the warp direction.

### 3.5. The Effect of the Structure on Bending Properties

Many studies [8,9,10,34] compared the performance of woven composites with different structures by controlling the material thickness and ensuring similar resin content/fiber volume fraction. For bending properties, Khatkar et al. showed that the flexural strength of 3D orthogonal composites was 50.7% higher than that of 2D plain composites. Therefore, this article compares the bending properties of 3DDAI armor material (unidirectional laying) and 2D plain weave laminated Kevlar/EP armor material by controlling the fabric surface density and resin content to achieve a similar thickness and material density. The specifications of the 3DDAI Kevlar/EP armor material and the 2D plain laminated Kevlar/EP armor material are shown in Table 8.

**Figure 13:**
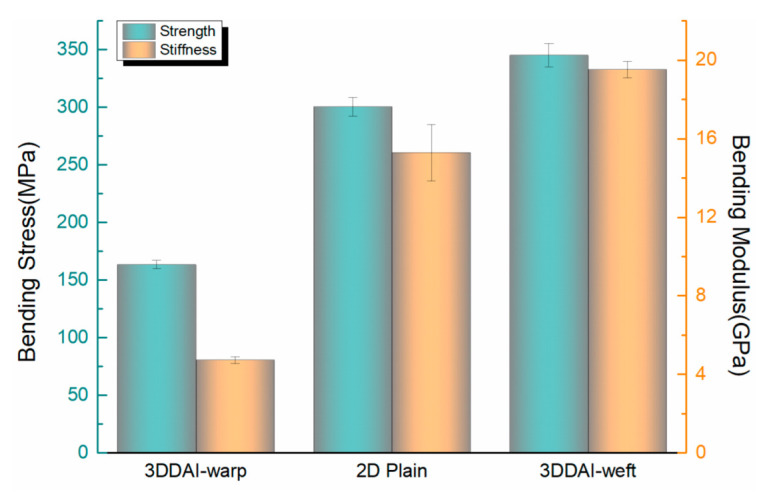
Comparison of the bending properties of the 3DDAI Kevlar/EP armor material and the 2D plain laminated Kevlar/EP armor material.

Figure 13 shows the comparison of the bending properties of the 3DDAI Kevlar/EP armor material and the 2D plain laminated Kevlar/EP armor material. As can be seen from the figure, the bending strength of 2D plain laminated Kevlar/EP armor materials was 304 MPa and the flexural modulus was 15.48 GPa, which was lower than that of the weft direction of 3DDAI Kevlar/EP armor materials but much higher than that of the warp direction of 3DDAI Kevlar/EP armor materials. This is due to the 3DDAI structure being through the buckled warp system in the thickness through the straightening weft system, intertwined into a three-dimensional structure. This structure improves the mechanics and structural integrity in the weft direction and thickness direction by sacrificing the mechanics of the warp direction.

**Figure 14:**
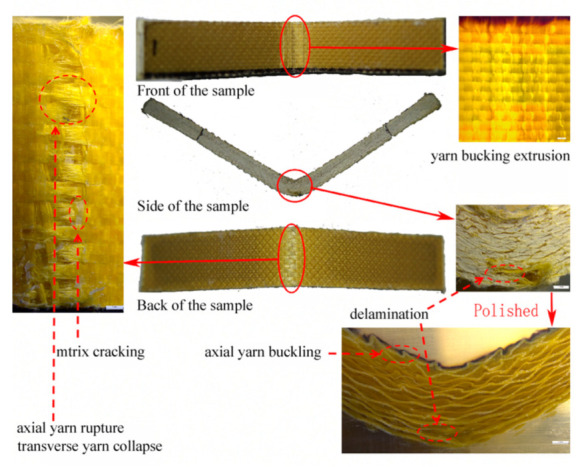
Bending failure morphology of 2D plain laminated Kevlar/EP armor material.

For further investigation, the bending failure morphology of the 2D plain laminated Kevlar/EP armor material in Figure 14 was compared with the bending failure of the 3DDAI Kevlar/EP armor material that was loaded in the weft direction as shown in Figure 5d. The analysis revealed that, in contrast to the oblique fracture path presented by the axial yarn of the 3DDAI Kevlar/EP armor material loaded along the weft direction, the axial yarn of the 2D plain laminated Kevlar/EP armor material presents the horizontal fracture path of the interwoven point area, and then fracture and delamination occur layer by layer.

This indicates that the 3DDAI Kevlar/EP armor material has better in-plane performance in the weft direction. Additionally, since the 3DDAI Kevlar/EP armor material along the weft direction of the axial yarn (weft yarn) was actually larger than the 2D plain weave laminated Kevlar/EP armor material, the bending performance of the 3DDAI Kevlar/EP armor material was superior to that of the 2D plain laminated Kevlar/EP armor material.

## 4. Conclusions

Based on the potential of 3DDAI Kevlar/EP armor material in the field of protection, the bending mechanics response of the 3DDAI Kevlar/EP armor material was carried out in terms of the number of fabric superimposed layers, resin content, laying method, and fabric weft density, and then compared with the bending performance of 2D plain laminated Kevlar/EP armor material. The following conclusions were obtained through research:

(1) When 3DDAI Kevlar/EP armor material is subjected to bending load, the upper and bottom layers of the material become the main carrying bodies, which has a greater impact on the initial stiffness and breaking strength of the material, respectively. Wherein the bending response of the 3DDAI Kevlar/EP armor material was nonlinear, the damage of the upper layer in the axial yarn and the damage of the matrix leads to the phenomenon of bending softening, and the fracture of the axial yarn in the bottom layer is the main cause of the material failure.
(2) Due to the particularity of the 3DDAI fabric structure, when the material’s warp and weft density are quite different, the utilization rate of the yarn and the strength will decrease. Furthermore, its loose structure needs to be appropriately increased in the resin content to prepare stable armor materials, where the appropriate range of resin content is 40%–49%. In addition, the 3DDAI Kevlar/EP armor can be prepared by the orthogonal laying method to improve the macroscopic mechanical properties of the material and effectively increase the fracture energy of the material.
(3) The 3DDAI Kevlar/EP armor material was in-plane anisotropic, and its bending performance along the weft direction was better than the 2D plain laminated material. Additionally, due to the penetration of the yarns in the thickness direction in the 3DDAI structure, even the lamination can effectively slow down the delamination of the material.

## Figures and Tables

**Table 1:** Kevlar fabric specifications.

Type	Fiber Type	Yarn Linear Density (Tex)	Warp Density (ends/cm)	Weft Density (picks/cm)	Thickness (mm)	Areal Weight (g/m^2^)
3DDI	Kevlar	111.11	10	43	0.80 ± 0.3	650 ± 3
3DDI	Kevlar	111.11	10	46	0.82 ± 0.3	662 ± 5
3DDI	Kevlar	111.11	10	50	0.85 ± 0.1	680 ± 6
Plain	Kevlar	111.11	9	9	0.21 ± 0.1	200 ± 2

**Table 2:** The detailed parameters of the 3DDAI Kevlar/EP armor material.

Number of Layers	Laying Method	Thickness (mm)	Weft Density (picks/cm)	Resin Content (%)	Fiber Volume Fraction (%)
1	Uni-Directional	1.26 ± 0.04	50	44.59	36.57
2	Uni-Directional	2.25 ± 0.04	50	43.64	42.78
3	Uni-Directional	3.16 ± 0.05	50	44.61	45.05
4	Uni-Directional	4.09 ± 0.05	50	44.59	46.36
4	Symmetrical	3.46 ± 0.08	50	34.27	54.92
4	Symmetrical	3.68 ± 0.04	50	36.75	52.71
4	Symmetrical	3.87 ± 0.05	50	40.39	48.94
4	Symmetrical	4.2 ± 0.06	50	44.61	46.12
4	Symmetrical	4.4 ± 0.06	50	48.59	43.38
4	2/2	4.06 ± 0.07	50	44.18	46.73
4	Orthogonal	4.11 ± 0.08	50	44.49	46.06
4	Orthogonal	4.26 ± 0.07	50	46.21	45.03
4	Orthogonal	4.06 ± 0.08	46	45.43	44.05
4	Orthogonal	4 ± 0.08	43	46.25	43.4

**Table 3:** The bending properties of materials with different stacking layers.

Stacking Layers	Bending Stress (MPa)				Bending Modulus (GPa)			
	Warp	CV%	Weft	CV%	Warp	CV%	Weft	CV%
1	67.7	5.0	200.0	10.0	2.31	1.4	9.83	13.7
2	126.0	1.2	312.0	2.8	2.96	3.5	14.02	6.0
3	169.4	1.5	356.0	2.5	4.41	4.2	18.61	2.4
4	165.9	2.0	345.3	2.6	5.75	1.4	19.52	1.9

**Table 4:** The bending properties of materials with different contents of epoxy resin.

Content of Epoxy Resin (%)	Bending Stress (MPa)			
	Warp	CV%	Weft	CV%
34.27	167.0	2.0	216.4	7.1
36.75	193.8	3.4	274.2	5.2
40.39	199.9	0.8	278.8	1.2
44.61	206.8	2.0	307.0	1.0
48.59	204.7	1.2	292.3	2.2

**Table 5:** Material fraction energy with various laying methods.

Laying Method		Linear Stage		Nonlinear Stage		Total
		Strain (mm/mm)	Area (J/mm^2^)	Strain (mm/mm)	Area (J/mm^2^)	Fraction Energy (J/mm^2^)
Unidirectional	Warp [0]_4_	0.0180	0.77	0.0774	8.22	9.00
	Weft [90]_4_	0.0142	1.70	0.0710	18.42	20.12
Orthogonal	Warp [90/0]_2_	0.0118	0.62	0.0694	11.06	11.68
	Weft [0/90]_2_	0.0164	1.28	0.1025	23.09	24.37
Symmetrical	Warp [0/90]_s_	0.0206	1.23	0.0802	11.02	12.25
	Weft [90/0]_s_	0.0102	0.80	0.0834	20.10	20.90
2/2	Warp [90_2_/0_2_]	0.0142	0.63	0.0514	6.15	6.78
	Weft [0_2_/90_2_]	0.0234	1.80	0.0988	18.64	20.43

**Table 6:** The bending properties of materials with different laying methods.

Laying Method		Bending Stress (MPa)	CV%	Bending Modulus (GPa)	CV%
Uni-Directional	Warp [0]_4_	165.9	2.0	4.79	1.4
	Weft [90]_4_	345.3	2.6	19.52	1.9
Orthogonal	Warp [90/0]_2_	215.7	2.4	11.52	4.2
	Weft [0/90]_2_	291.8	2.7	10.61	3.7
Symmetrical	Warp [0/90]_s_	207.0	2.0	6.67	3.4
	Weft [90/0]_s_	307.0	1.0	17.32	1.3
2/2	Warp [90_2_/0_2_]	206.8	8.8	8.79	3.3
	Weft [0_2_/90_2_]	266.2	2.1	8.23	0.9

**Table 7:** The bending properties of materials with different weft densities.

Weft Density (picks/cm)	Bending Stress (MPa)				Bending Modulus (GPa)			
	Warp	CV%	Weft	CV%	Warp	CV%	Weft	CV%
43	220.8	2.7	267.6	1.6	10.42	4.4	8.81	2.0
46	204.7	2.1	260.5	2.0	10.66	2.6	9.97	2.1
50	194.6	4.1	238.7	3.4	11.00	6.2	10.71	4.6

**Table 8:** 3DDAI armor material, 2D plain laminated armor material parameters.

Type	Resin Content (%)	Fabric Areal Weight (g/m^2^)	Thickness (mm)	Density (g/cm^3^)
2D plain (14 pieces)	43.08	2800	3.87	1.26
3DDAI (4 pieces)	44.11	2720	4.09	1.21

## Data Availability

Data is contained within the article.
